# Quantification and characterization of biological activities of glansreginin A in black walnuts (*Juglans nigra*)

**DOI:** 10.1038/s41598-023-46134-8

**Published:** 2023-11-01

**Authors:** Khanh-Van Ho, Hsien-Yeh Hsieh, Anuradha Roy, Sarah Foote, Peter McDonald, Mark V. Coggeshall, Hideyuki Ito, Zhentian Lei, Lloyd W. Sumner, George C. Stewart, Chung-Ho Lin

**Affiliations:** 1 Center for Agroforestry, School of Natural Resources, University of Missouri, Columbia, MO USA; 2 Department of Chemistry, University of Missouri, Columbia, MO USA; 3 Molecular Imaging and Theranostics Center, University of Missouri, Columbia, MO USA; 4 Department of Post-harvest Technology, Can Tho University, Can Tho, Vietnam; 5 High Throughput Screening Laboratory, University of Kansas, Lawrence, KS USA; 6 CEVA Biomune, Lenexa, KS USA; 7 Northern Research Station, USDA Forest Service, West Lafayette, IN USA; 8 Faculty of Health and Welfare Science, Department of Nutritional Science, Okayama Prefectural University, Okayama, Japan; 9 Metabolomics Center, University of Missouri, Columbia, MO USA; 10 Department of Biochemistry, Bond Life Sciences Center, University of Missouri, Columbia, MO USA; 11 Department of Veterinary Pathobiology, Bond Life Sciences Center, University of Missouri, Columbia, MO USA

**Keywords:** Mass spectrometry, Assay systems

## Abstract

Glansreginin A has been reported to be an indicator of the quality of walnuts (*Juglans* spp.). However, bioactive properties of glansreginin A have not been adequately explored. In the present study, we quantified concentrations of glansreginin A in black walnuts (*Juglans nigra*) using high performance liquid chromatography-tandem mass spectrometry (HPLC–MS/MS) and performed an array of in vitro bioassays to characterize biological activities (e.g., antibacterial, antioxidant, anticancer capacities) of this compound. Results from HPLC–MS/MS analysis indicated that glansreginin A was presented in all 12 black cultivars examined and its contents were variable among black walnut cultivars, ranged from 6.8 mg/kg (Jackson) to 47.0 mg/kg (Hay). Glansreginin A possessed moderate antibacterial activities against Gram-positive pathogens (*Staphylococcus aureus* and *Bacillus anthracis*). This compound exhibited no antioxidant activities, did not induce the activity of antioxidant response element signaling pathways, and exerted no antiproliferative effects on tumorigenic alveolar epithelial cells and non-tumorigenic lung fibroblast cells.

## Introduction

**Figure 1:**
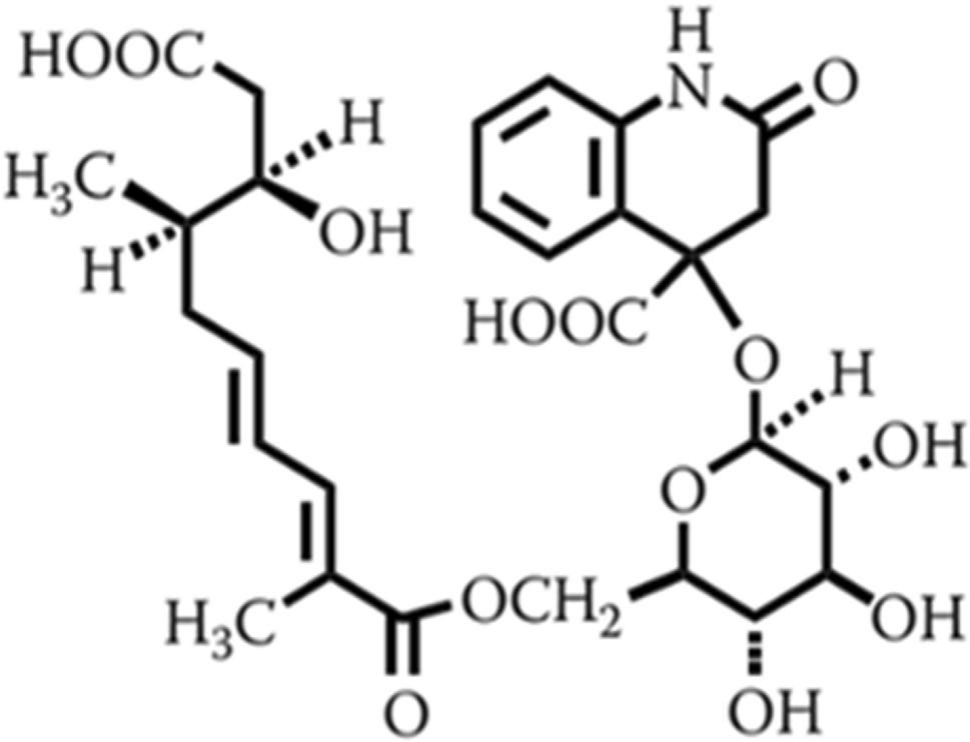
Glansreginin A.

Glansreginin A, a dicarboxylic acid derivative (Fig. 1), has been documented as an indicator component of the quality of English walnuts (*Juglans regia*)^1,2^. This compound has been detected as a major constituent in the kernel extracts of English walnut which is the most important species in the genus of *Juglans* for nut production^1,3,4^. Glansreginin A has been putatively identified in the kernels of pecan (*Carya illinoinensis*) and hazelnut (*Corylus avellana*)^5,6^. This compound has not been found in other common tree nuts including almond (*Prunus dulcis*), pistachio (*Pistacia vera*), cashew nut (*Anacardium occidentale*), and macadamia nut (*Macadamia* sp.)^1^.

Limited information on the biological functions of glansreginin A is available. Ito et al.^2^ evaluated antioxidant capacities of glansreginin A by determining superoxide dismutase (SOD)-like activity and DPPH (2,2-diphenyl-1-picrylhydrazyl) radical scavenging effects of this compound. The authors found that glansreginin A had no antioxidant activities in both assays. Haramiishi et al.^1^ documented neuroprotective effects of glansreginin A via anti-inflammation in the brain using lipopolysaccharide (LPS)-induced inflammatory model mice. This group reported that LPS-induced abnormal behavior and LPS induced hyper-activation of microglia in the hippocampus were significantly reduced in the LPS-injected mice with oral administration of glansreginin A (50 mg/kg for 8 days) in comparison to those without ingestion of glansreginin A.

Our previous study indicated the possibility of the presence of glansreginin A in the kernels of black walnuts (*Juglans nigra*)^7^, another common *Juglans* species. Black walnut is one of the most economically valuable hardwood species^8^. This native plant species is a highly value tree for edible nut production in North America^9^ as the second highest produced walnut nut in the United States^4^. Glansreginin A has been tentatively identified in a black walnut cultivar (Mystery) by comparing high-resolution mass spectral data of this compound generated from the kernel extract of Mystery with the known spectral data of glansreginin A in literature^7^. Since glansreginin A is not commercially available, validation and quantification of concentrations of glansreginin A in black walnuts were not further investigated in Ho et al.^7^. In the present study, we validated the presence of glansreginin A and determined its contents in 12 black walnut cultivars using ultra-high performance liquid chromatography with high-resolution mass-spectrometry (UHPLC-HRMS) and liquid chromatography-tandem mass spectrometry (HPLC–MS/MS) analyses, respectively. Following the quantitative analysis, we characterized several biological activities (antibacterial, antioxidant, anticancer capacities) of purified glansreginin A using high-throughput in vitro bioassays.

## Results

### Validation the presence of glansreginin A in black walnuts

**Figure 2:**
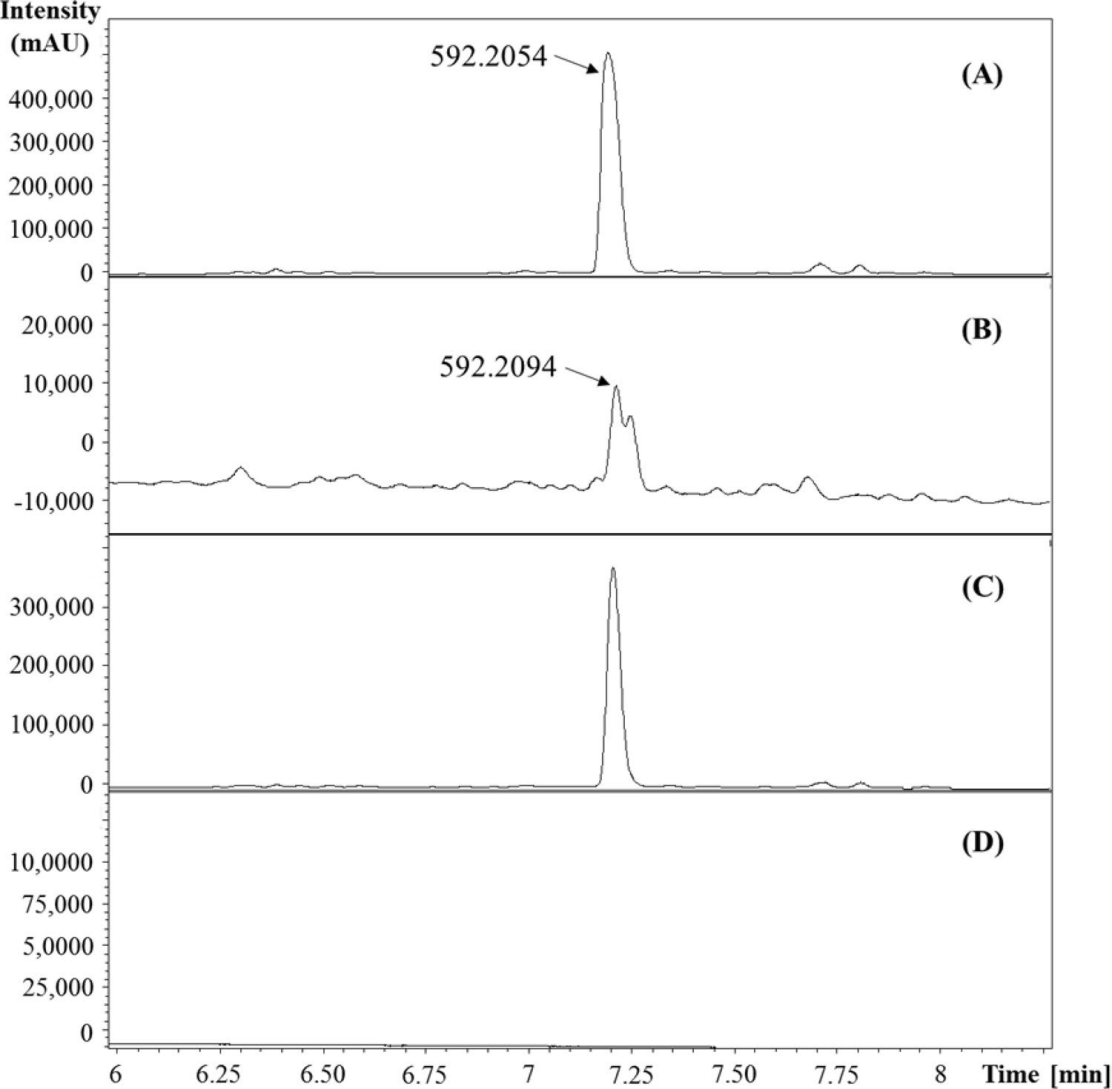
UHPLC UV chromatogram (267 nm) of glansreginin A (**A**), the bioactive fraction in Mystery (**B**), glansreginin A spiking to the bioactive fraction (**C**), the solvent (**D**).

**Figure 3:**
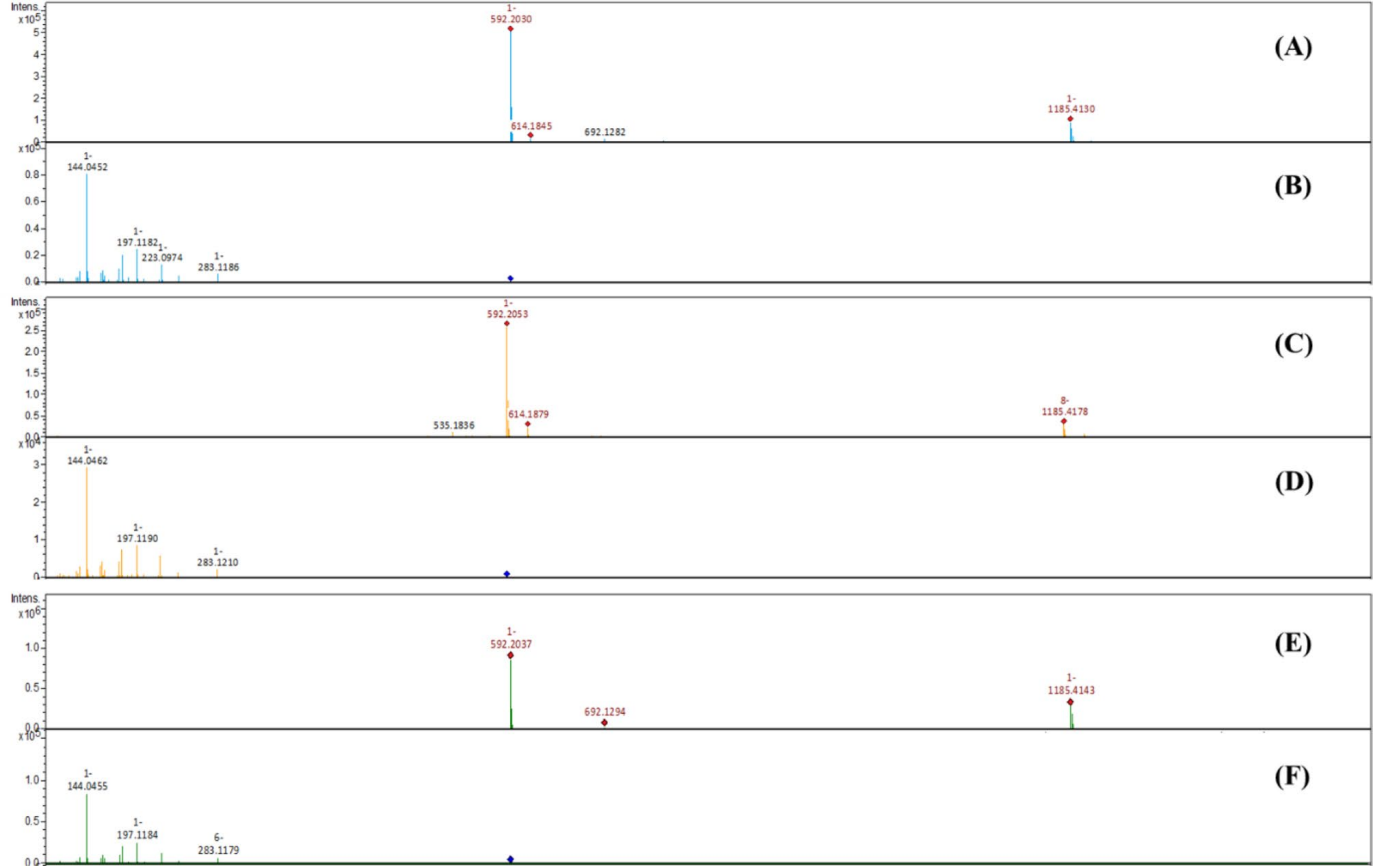
Mass spectra of purified glansreginin A (**A**, Mass spectrum; **B**, MS/MS spectrum of m/z 592.20), the bioactive fraction in Mystery (**C**, Mass spectrum; **D**, MS/MS spectrum of m/z 592.20), glansreginin A spiking to the bioactive fraction (**E**, Mass spectrum; F, MS/MS spectrum of m/z 592.20), detected by UHPLC-MS/MS. The MS/MS spectrum of the bioactive fraction (**D**) matches to that of the purified glansreginin A (**B**), confirming the identity of the bioactive fraction as glansreginin A.

**Figure 4:**
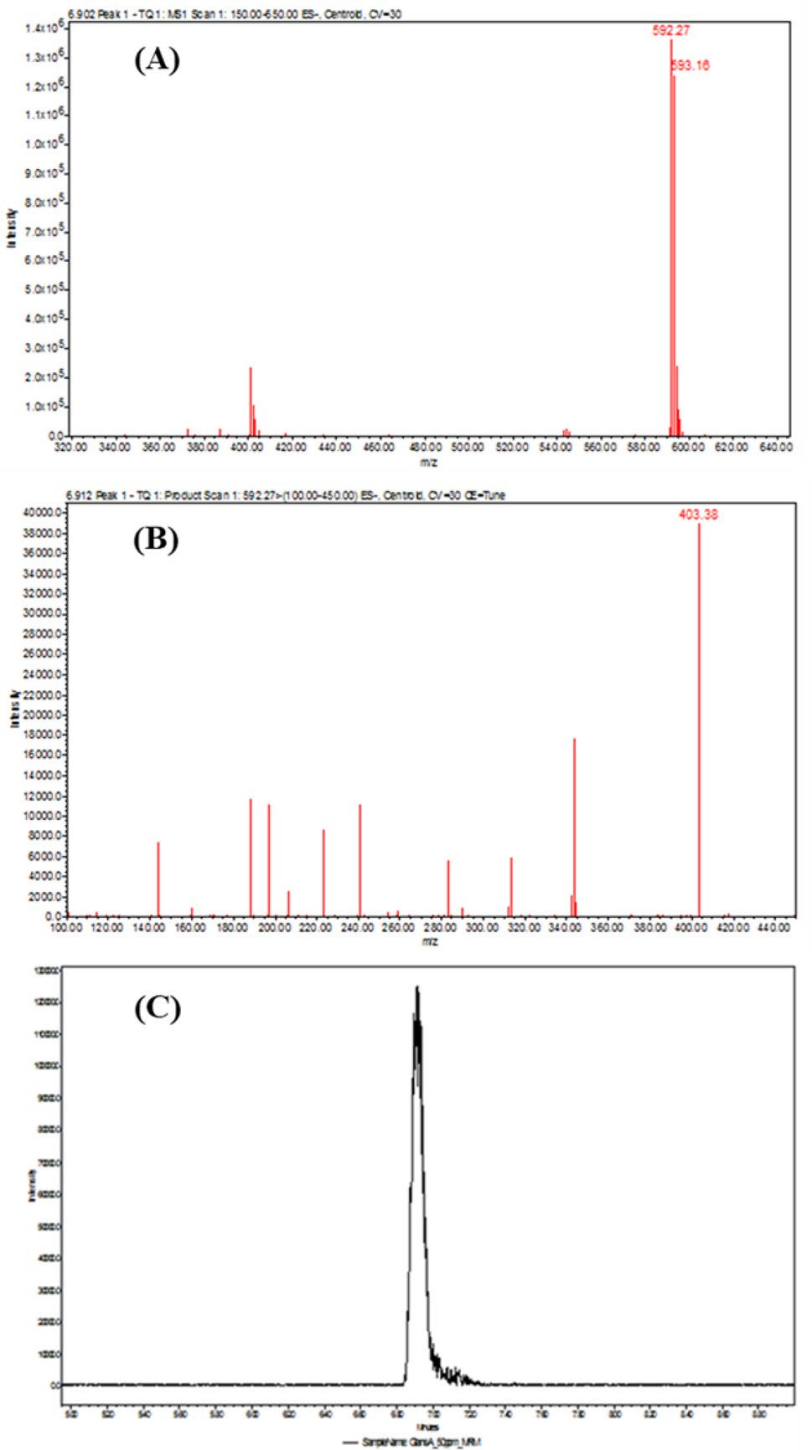
The full mass spectra of molecular ion [M-H]^−^. (**A**) and product ions (**B**) of glansreginin A. The LC–MS/MS ion chromatogram of m/z 403 with 50 ppm of glansreginin A (**C**).

The purified glansreginin A derived from English walnut kernels, the bioactive fraction of a black walnut cultivar (Mystery), and the sample with the purified compound spiking to the bioactive fraction had UHPLC chromatograms at a wavelength of 267 with the same major peak (Fig. 2). Glansreginin A in the active fraction of Mystery at retention time at 7.17 min was the same as the purified compound at retention time at 7.13 min. The tandem MS and MS/MS spectra of the major peak in the purified compound, the bioactive fraction of Mystery, and the sample including the purified compound and the bioactive fraction were similar (Figs. 3 and 4).

### Quantification of glansreginin A in black walnuts

Glansreginin A was detected in the kernels of all 12 black walnut cultivars. The concentrations of this compound varied among the examined cultivars (Table 1). This compound was found to be at the highest amount in Hay (47.0 ± 4.0 mg/kg), followed by Emma (38.1 ± 5.3 mg/kg) and Thomas (29.1 ± 4.3 mg/kg), Schessler (25.3 ± 4.3 mg/kg), Hare (24.2 ± 5.2 mg/kg), and Bowser (20.4 ± 4.0 mg/kg). Compared to these cultivars, the contents of glansreginin A in other tested cultivars were significantly lower. Specifically, the concentrations of glangreginin A in Surprise, Mystery, Davidson, South Fork and Jackson were 17.3 ± 7.6, 14.5 ± 1.4, 13.1 ± 3.3, 10.9 ± 3.0, 6.8 ± 2.3 mg/kg, respectively.

**Table 1:** Contents of glansreginin A (mg/kg of dry weight) in kernels of 12 black walnut cultivars.

No.	Black walnut cultivar	Content (mg/kg of dry weight)*
1	Bowser	20.4 ± 4.0 CDE
2	Davidson	13.1 ± 3.3 FG
3	Emma	38.1 ± 5.3 B
4	Hay	47.0 ± 4.0 A
5	Hare	24.2 ± 5.2 CD
6	Jackson	6.8 ± 2.3 H
7	Mystery	14.5 ± 1.4 FG
8	Schessler	25.3 ± 4.3 C
9	Sparks	19.1 ± 3.5 DE
10	South Fork	10.9 ± 3.0 GH
11	Surprise	17.3 ± 7.6 EF
12	Thomas	29.1 ± 4.3 C

*Means with bars followed by different letters are significantly different (*p* < 0.05). Means ± SD (n = 3).

### Bioactive activities of glansreginin A

#### Antibacterial activities

**Figure 5:**
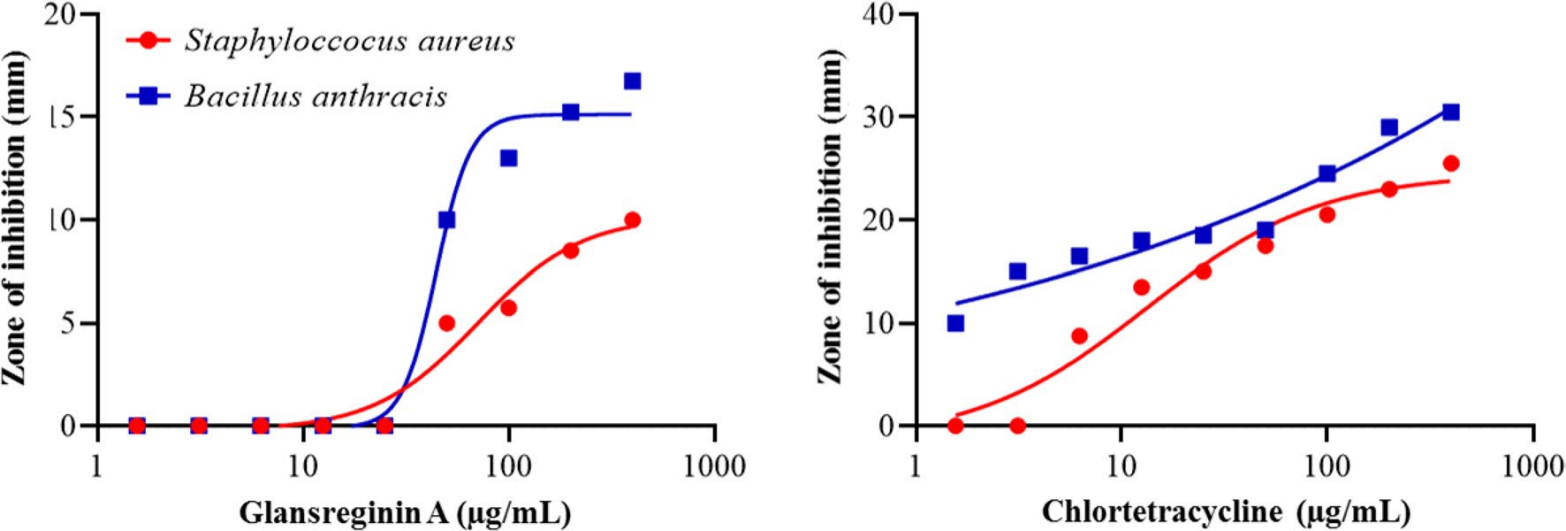
Zones of inhibition of glansreginin A and chlortetracycline for *Bacillus anthracis* and *Staphylococus aureus*. The diameter of the wells in agar-well diffusion assays was 4.5 mm. The concentrations of the test compounds with zones of inhibition > 4.5 mm had inhibitory effects against the examined bacteria.

The minimum inhibitory concentration (MIC) values of glansreginin A for *B. anthracis* and *S. aureus* were 50 and 100 µg/mL, respectively, whereas the MIC values of chlortetracycline for *B. anthracis* and *S. aureus* were < 1.56 and 6.25 µg/mL, respectively. Figure 5 shows the zone of inhibition of glansreginin A and chlortetracycline that were positively correlated with increases in the concentrations of these compounds.

#### Antioxidant activities and ARE activation

**Figure 6:**
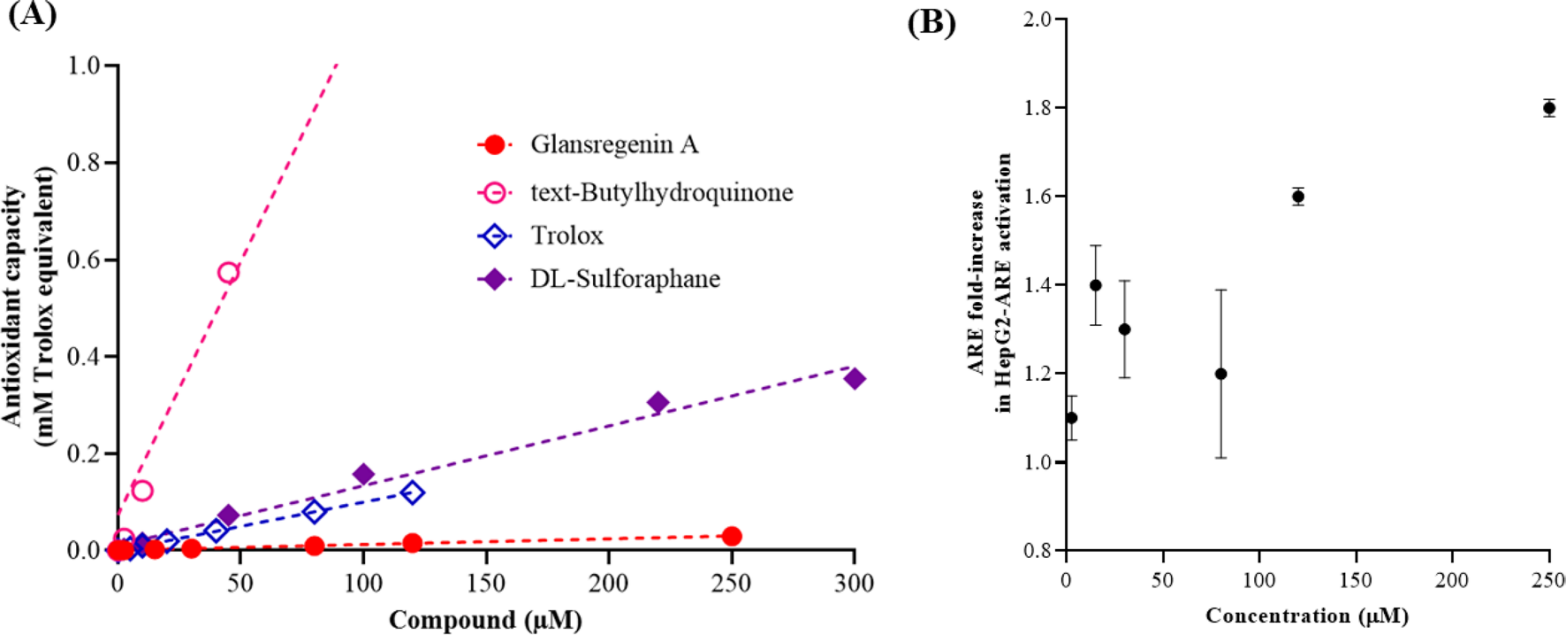
Antioxidant activities of glansreginin A. Total antioxidant activity (**A**), antioxidant response element activation activities of glansreginin A in HepG2-ARE cells (**B**).

Linear regression models of each compound tested were generated and compared with the Trolox control. The R^2^ values of all models were high (> 0.98), indicating that these models were reliable (Suppl. Table 1). Compared with the control (Trolox), the total antioxidant capacity of glansreginin A was significantly lower, whereas TBHQ and DL-sulforaphane exhibited higher total antioxidant capacity (Fig. 6A). The fold-increase over Trolox of glansreginin A was 0.12 which is 8.3 times lower compared with Trolox. TBHQ and DL-sulforaphane had the fold-increase over Trolox of 10.44 and 1.23, respectively.

#### Antioxidant response element (ARE) activation

Glansreginin A at tested concentrations was not toxic to the HepG2-ARE cells. The ARE fold-increase in HepG2-ARE activation of glansreginin A at tested concentrations relative to the control was < 2 (Fig. 6B). Glansreginin A exhibited a very low total antioxidant capacity and with the minimal ARE fold-increase in HepG2-ARE, this compound would not be considered to possess ARE induction activity.

#### Anticancer activities

**Figure 7:**
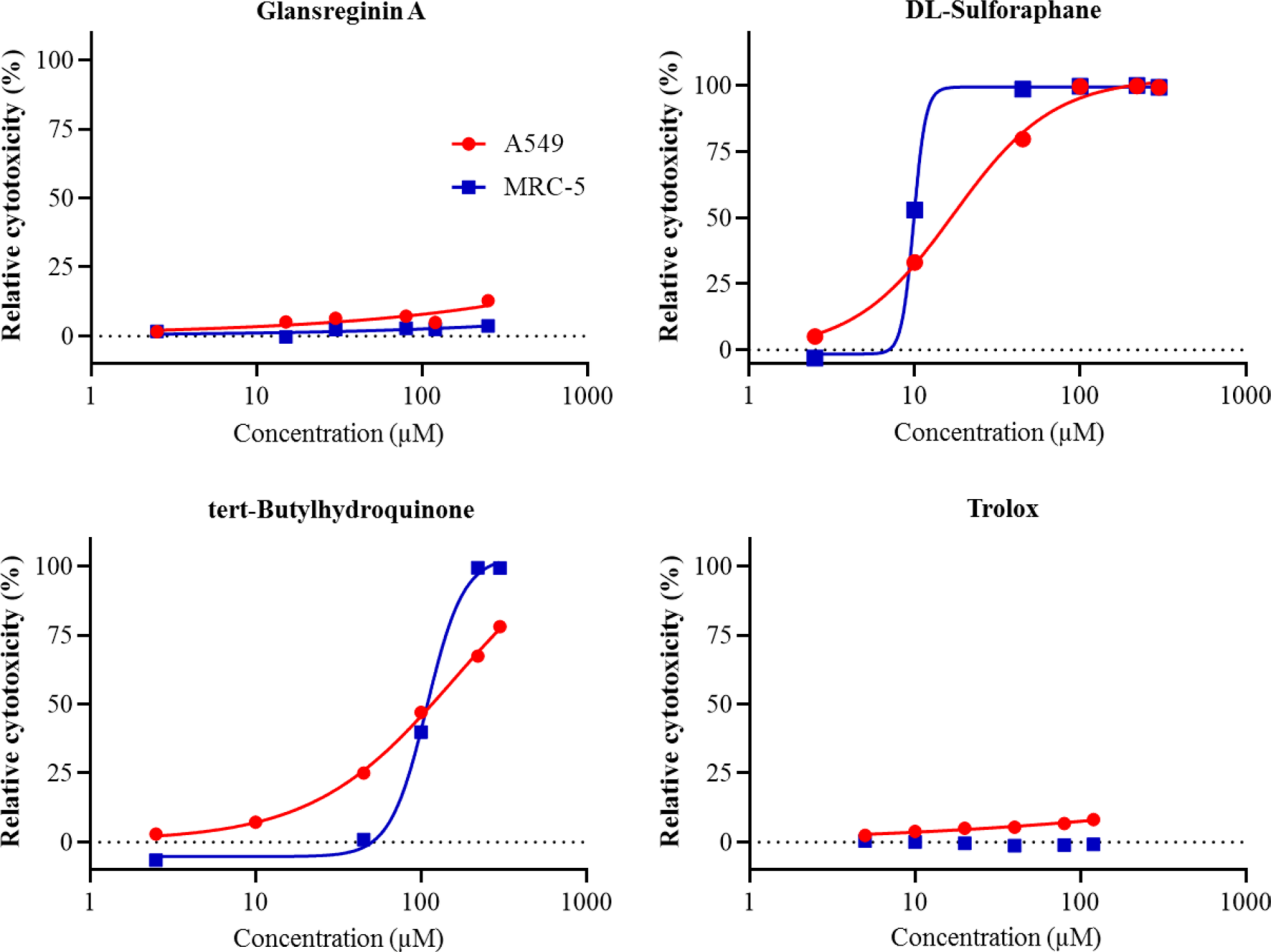
Cytotoxicity (%) of glansreginin A, Trolox, text-Butylhydroquinone, DL-Sulforaphane in tumorigenic alveolar epithelial cells (A549) and non-tumorigenic lung fibroblast cells (MRC-5). Data are expressed as percentages of cytotoxicity in A549 and MRC-5 cells treated with DMSO and the compounds compared with the corresponding vehicle controls that were A549 and MRC-5 cells treated with 0.35% DMSO only.

The cytotoxic effects of glansreginin A on the growth of A549 and MRC-5 cells were determined using cell viability assays. A reduction in cell viability or cell number results in reduced luminescence. Results from the assays showed that the vehicle control (DMSO at 0.35%) exhibited no effect on cell number or viability in the A549 and MRC-5 cells, revealing that a reduction in luminescence in the presence of the tested compounds would indicate a toxic effect of the compounds rather than the vehicle. The IC_50_ values of glansreginin A were higher than 250 for both A549 and MRC-5 cells (Suppl. Table 2), while the IC_50_ values of Trolox were higher than 120 for the tested cell lines. The positive control (DL-sulforaphane) and TBHQ had IC_50_ values for A549 and MRC-5 cells were 16.87 and 9.27, and 139.2 and 108.5 µM, respectively (Fig. 7).

## Discussion

Glansreginin A levels were documented to correlate with the kernel quality of English walnuts (*J. regia*)^1^. This compound likely contributes to the biological activities of black walnuts (*J. nigra*) which have been demonstrated as a promising natural source for the medicinal and pharmaceutical industries^7,10–12^. In this study, the contents of glansreginin A in the kernels of 12 black walnut cultivars selected for nut production were identified using HPLC–MS/MS analysis and the biological properties of glansreginin A (antibacterial, antioxidant and anticancer activities) were characterized via an array of in vitro bioassays. The exploration of the biological functions of glansreginin A, a signature compound in walnuts could promote the development of novel applications of black walnut and its by-products in cosmetic and pharmaceutical industries. This could result in profitable value-added products of black walnut obtained from the abundant, low-value, renewable materials from black walnut, which potentially increases the sustainability of the black walnut agro-industry.

Our previous study has putatively identified glansreginin A in the bioactive fraction in the kernel extract of a black walnut cultivar, Mystery based on the mass spectral information of this compound available in MetFrag metabolite database^7^. In the current study, glansreginin A was successfully validated in the bioactive fraction from Mystery using a purified reference glansreginin A derived from English walnut. The presence of the glansreginin A was confirmed by both UHPLC-HRMS and HPLC–MS/MS. Our results also revealed that the presence of glansreginin A in all black walnut cultivars examined. Its concentrations varied among the tested cultivars, ranged from 6.8 mg/kg (Jackson) to 47.0 mg/kg (Hay). It’s interesting that antibacterial activities of the kernel extracts are not positively correlated with the amount of glansreginin A in their cultivars. Our previous study demonstrated that Mystery had the highest antibacterial activities against a Gram-positive bacterium (*S. aureus*), whereas Mystery was found to contain a moderate amount of glansreginin A (17.7 mg/kg) among the examined cultivars. This finding suggests that the antibacterial activities of the kernel extracts could result from the combination effects of several bioactive compounds in the kernel extracts in which glansreginin A is one of these components.

Black walnuts have been reported to contain a wealth source of polyphenols such as syringic acid, ellagic acid, and hydrolysable tannins^13–16^. The contents of phenolic compounds were found to be highly variable. Ellagic acid has been reported as one of the most abundant polyphenols in black walnuts. Figueroa et al.^15^ reported the concentrations of ellagic acid of 217.3–704.7 mg/kg, whereas its concentrations in a range of 11.4–97.7 mg/kg in other studies. Vu et al.^16^ characterized phenolic compounds in black walnut and reported a variation in the concentrations of 16 phenolic compounds detected among and within 11 black walnut cultivars. Ellagic acid was found as the most abundant phenolic compounds in black walnuts, and its concentrations ranged from 9 mg/kg (Tomboy) to 72 mg/kg (Surprise), whereas other phenolic compounds were mostly less than 10 mg/kg in the black walnut cultivars evaluated^16^. Compared to the known phenolic compounds in black walnut, glansreginin A was presented at a relatively high levels in the kernels of black walnuts.

In order to identify an indicator compound in English walnuts (*J. regia*), Haramiishi et al.^1^ compared the kernel extract derived from English walnuts with those of other common tree nuts (i.e., almond, pistachio, hazelnut, cashew nut, pecan nut, and macadamia nut) using HPLC analyses. The authors reported that glansreginin A was only present in the English walnut extract and was not observed in the extracts derived from any other common tree nuts. This group further reported the presence of glansreginin A in every commercial English walnut and suggested that glansreginin A can be used as the indicator component in walnuts. Previously, Ito et al. ^2^ extracted and isolated glansreginin A from an English walnut cultivar, Chandler and obtained 94.2 mg/kg of glansreginin A of dry weight of the kernels. Later, the concentrations of glansreginin A were determined in several English walnut cultivars using chlorogenic acid or ellagic acid as a referenced standard and estimated its concentrations (fresh weight of the kernels) in a range of 76.3 mg/kg (Chandler)–847.7 mg/kg (Fernette)^3,17^. Compared to English walnuts, black walnuts (*Juglans nigra*) contain lower levels of glansreginin A with its concentrations in the kernels ranging from 6.8 to 47.0 mg/kg (dry weight). Noticeably, glansreginin A has been putatively identified in hazelnuts and pecan nuts with the reference to the known mass spectral data of this compound^5,6^. Using a gallic acid calibration curve, Slatnar et al.^6^ estimated the concentrations of glansreginin A in the kernels of four hazelnut cultivars in a range of 18 mg/kg (Istrska dolgoplodna leska)–110.9 mg/kg (Fertile de Coutard).

This study added to the limited number of studies characterizing the biological functions of glansreginin A. So far, only a few studies had reported biological functions of glansreginin A. Our results revealed that glansreginin A exerts moderate antibacterial activities against both Gram-positive bacteria (*B. anthracis* and *S. aureus*) tested. MIC values of glansreginin A for *B. anthracis* and *S. aureus* were 50 and 100 µg/mL, respectively, which were higher than those obtained for chlortetracycline. In respect to other biological activities, glansreginin A exhibited no antioxidant activities and no antiproliferative effects on tumorigenic alveolar epithelial cells and non-tumorigenic lung fibroblast cells. Previously, Haramiishi et al.^1^ demonstrated anti-inflammatory activity of glansreginin A via neuroprotective effect on the brain of LPS-injected mice. The LPS-injected mice consumed glansreginin A (50 mg/kg for 8 days) had a significant reduction of LPS-induced abnormal behavior and LPS induced hyper-activation of microglia in the hippocampus, compared to those without the consumption of glansreginin A. Glansreginin A has also been previously reported to possess no antioxidant activities determined by SOD like activity and DPPH radical scavenging effects^2^.

## Material and methods

### Black walnut material

The in-shell nuts of 12 black walnut cultivars including Bowser, Davidson, Emma, Hay, Hare, Jackson, Mystery, Schessler, Sparks 147, South Fork, Surprise, and Thomas were collected in November 2017 at the University of Missouri, Horticulture and Agroforestry Research Center (New Franklin, MO, USA). The black walnut trees were well cultivated for nut production with typical practices (e.g., lime, pruning, fertilization, weed control, and pest and disease management)^18^. The nuts were obtained at their technological maturity when the mature nuts had fallen to the ground^19^. After collection, these nuts were husked mechanically and dried at 24 °C for 15 days and the hulled nuts were then stored at − 20 °C until analysis.

### Sample extraction

The hulled nuts were cracked manually and then the shells were removed to collect peeled kernels of black walnuts. The peeled kernels were homogenized using a coffee grinder and were extracted as described previously^10^. Briefly, the homogenized samples (100 g, dry weight basis, 20–30 mesh) were extracted in 400 ml methanol twice and then sonicated at 10 °C for 60 min, followed by centrifugation for 10 min at 4550 g. Several extractions of different peeled kernels derived from each black cultivar were performed and utilized in at least triplicate in quantitative analyses and in vitro assays. The methanolic extracts were filtered through a 0.2 µm Whatman filter paper (GE Healthcare, Chicago, IL, USA) under SPE Vacuum Manifold (Visiprep™ SPE Vacuum Manifold, Sigma-Aldrich, St. Louis, MO, USA). The resulting supernatant was then collected and stored at − 20 °C until analysis.

### Glansreginin A

Our previous study has putatively identified the presence of glansreginin A in the bioactive fraction in the kernel extract of a black walnut cultivar, Mystery by comparing mass spectral data of this compound in the extract of Mystery with the known spectral data reported in the MetFrag metabolite database (msbi.ipb-halle.de/MetFragBeta/)^7^. Since glansreginin A is not commercially available, concentrations of this compound in black walnut kernels were not previously identified^7^. In the present study, glansreginin A was extracted, isolated, and purified from the peeled kernels of English walnut, a closely related species of black walnut as described previously^2^. This purified reference standard has been confirmed by direct comparison with authentic specimens^2^. The purified compound was formed as a pale-yellow amorphous powder. The purified glansreginin A was utilized for the validation and the determination of the contents of glansreginin A in 12 black walnut cultivars as well as for identification of its biological activities (antibacterial, antioxidant and anticancer capacities).

### Validation of glansreginin A in black walnuts

The presence of glansreginin A in Mystery was validated by comparing the retention time and mass spectra of this compound in the Mystery fraction with the mass spectra of the purified glansreginin A. Mass spectral data of the purified compound, the black walnut extracts, and the blank (solvent control) were acquired and compared. The mass spectra of all samples (2 µL per injection) were generated by an ultra-high performance liquid chromatography (UHPLC) system coupled to a maXis impact quadrupole-time-of-flight high-resolution mass spectrometer (Q-TOF) (Bruker Co., Billerica, MA, USA) as described previously^7^. The system was operated in a negative electrospray ionization mode with the nebulization gas pressure at 43.5 psi, dry gas of 12 L/min, dry temperature of 250 °C and a capillary voltage of 4000 V. The mass spectral data were collected at retention time (rt) of 7.18 min and *m*/*z* of 592.2043.

### Quantification of glansreginin A in 12 black walnut varieties

The contents of glansreginin A in the kernels of 12 black walnut cultivars were determined using a Waters Alliance 2695 High Performance Liquid Chromatography (HPLC) system coupled with Waters Acquity TQ triple quadrupole mass spectrometer (MS/MS) as described previously^20,21^. The system was operated in negative electrospray ionization mode (ES^−^) with the nebulization gas pressure at 43.5 psi, dry gas of 12 L/min, dry temperature of 250 °C and a capillary voltage of 1500 V. The compounds in the kernel extracts of black walnut (20 µL per injection) were separated by a Phenomenex Kinetex C18 reverse-phase column (100 × 4.6 mm; 2.6 µm particle size, Torrance, CA, USA). The MS/MS system was operated in the multi-reaction monitoring (MRM) mode with optimized collision energy. Waters IntelliStart optimization software was used to optimize collision, ionization energy, MRM transition ions (molecular and product ions, 592.6 → 403/343), capillary and cone voltage, desolvation gas flow, and collision energy. Assessment of the sensitivity of the analytical method was determined by calculating limit of detection (LOD) and limit of quantification (LOQ) of glansreginin A that were identified by employing signal-to-noise ratios of three and ten. The concentrations of glansreginin A in the extracts in triplicate were determined based on a standard curve for this compound generated using the purified glansreginin A at 8 concentrations (0.1, 0.5, 1, 5, 10, 25, 50 and 100 ppm) in six replicates.

### Exploration of bioactive activities of glansreginin A

#### Antibacterial activity

The antibacterial capacities of glansreginin A were evaluated on the growth of two Gram-positive bacteria (*S. aureus* strain RN 6390 and *Bacillus anthracis* Sterne) using agar-well diffusion assay as described by Holder and Boyce^22^. These bacteria were cultured on Luria–Bertani (LB) agar plates as described previously^7^. Briefly, the bacteria were cultured in 5 mL LB broth at OD_600_ of 0.02 in a shaker at 37 °C. Once the culture reached an OD_600_ of 0.1, the bacteria were swab-inoculated onto LB agar plates. Subsequently, a cork borer was used to punch the surface of the agar and the cutting agars were removed to create punched wells (4.5 mm in diameter, 20 wells per plate) in the plates. The compounds tested (10 µL per well) were pipetted into the punched wells and the plates then incubated under aerobic condition at 37 °C for 16 h. Glansreginin A and chlortetracycline (Sigma-Aldrich), a known bacteriostatic antibiotic, were tested at 9 final concentrations in 2 × serial dilution, ranging from 1.56 to 400 µg/mL. The diluting solvent (DMSO at 100%) served as vehicle controls. Each treatment was replicated at least 4 times in different LB agar plates. MIC values of glansreginin A and chlortetracycline were identified for each bacterium tested. The diameters of inhibition zones were measured by a ruler, with an accuracy of 0.5 mm. Each inhibition zone was measured three times. The diameter of the wells in agar-well diffusion assays was 4.5 mm. The concentrations of the test compounds with zones of inhibition > 4.5 mm had inhibitory effects against the examined bacteria.

#### Total antioxidant capacity

The total antioxidant capacity of glansreginin A was determined by a total antioxidant capacity (TAC) colorimetric assay kit (K274-100, BioVision, CA, USA), according to the manufacturer’s instructions^12^. Glansreginin A was evaluated for its total antioxidant activity at 7 concentrations of 0, 2.5, 15, 30, 80, 120, and 250 µM, whereas DL-sulforaphane (Sigma-Aldrich) and tert-butylhydroquinone (TBHQ, Sigma-Aldrich), which were used as positive controls, were tested at 7 concentrations of 0, 2.5, 10, 45, 100, 220, and 300 µM. Trolox was included to standardize the antioxidant capacity, as recommended by the manufacturer. For the assays, the tested compounds were added to 384-well plates, followed by an addition of 2.5 mM Cu^2+^ working solution (12.5 µL/ well) into the sample wells. The plates were incubated for 1.5 h at room temperature. Subsequently, the absorbance of the samples was then read at 570 nm using a microplate reader (Enspire, Perkin Elmer Inc., Waltham, MA, USA). The total antioxidant capacity of the tested compounds was calculated from a standard curve of Trolox at 7 concentrations (0, 5, 10, 20, 40, 80, and 120 µM) and was expressed as Trolox equivalent (µM).

#### Antioxidant response element (ARE) activation

The influence of glansreginin A on the activity of ARE signaling pathways that regulate expression of genes encoding over 250 antioxidant and detoxification proteins^23^ was determined using Steady-Glo® Luciferase assay system (E2510, Promega, Madison, WI, USA) as described previously^12^. Brieflty, an Nrf2 ARE reporter HepG2 cell line (HepG2-ARE), a stably transfected liver cell line expressing a firefly luciferase gene under the control of the ARE, was purchased from BPS Bioscience (San Diego, CA, USA). The HepG2-ARE cells were grown in modified Eagle’s medium (MEM) supplemented with GlutaMAX, 10% fetal calf serum (FBS) and 600 µg/mL Geneticin (Thermo Fisher Scientific, Waltham, MA, USA), and maintained at 37 °C in a humidified incubator with 5% CO_2_. The HepG2-ARE cells were seeded at a density of 10^4^ cells/well in 384-well plates that contain the complete media (50 µL per well) using a Multidrop Combi dispenser (Thermo Fisher Scientific, Waltham, MA, USA). The plate cultures were incubated at 37 °C in a 5% CO_2_ humidified incubator for 20 h. Subsequently, the HepG2-ARE cells were incubated with glansreginin A at 7 concentrations (as described above) for 18 h. The known ARE activator TBHQ at 7 concentrations (0–300 µM) was used as a positive control and the cells treated with 0.35% DMSO and without the tested compounds served as a vehicle control. The cells without additions of DMSO and tested compounds were used for measuring the background luminescence. Steady-Glo® luciferase assay reagent (Promega) was added at 25 µL per well for 30 min to measure the reporter activity using the Multidrop Combi dispenser (Thermo Fisher Scientific). An Enspire microplate reader (Perkin Elmer Inc.) was used to read the luminescence intensities of the 384-well plates. The ARE fold induction of the compounds was calculated by dividing the luminescence of the treatment group by the specific luminescence of the vehicle control.

#### Cell proliferation assays

The anticancer activities of glansreginin A were identified by evaluating the effects of this compound on the growth of the tumorigenic alveolar epithelial cells (A549) and non-tumorigenic lung fibroblast cells (MRC-5) using CellTiter-Glo® cell viability assay kit (G7571, BioVision, CA, USA). The A549 and MRC-5 cells were purchased from the American Type Culture Collection (ATCC) (CCL-185 and CCL-171, ATCC, Manassas, VA, USA). These cells were grown in RPMI medium supplemented with 10% FBS and maintained at 37 °C in a humidified incubator with 5% CO_2_. The A549 and MRC-5 cells at densities of 8 × 10^3^ and 3 × 10^3^ cells per well, respectively, were seeded in 384-well plates and the plates were incubated at 37 °C in a 5% CO_2_ humidified incubator. Subsequently, the cultures were then treated with glansreginin A and DL-sulforaphane (a known antiproliferative agent as a positive control) at 7 final concentrations (0–300 µM) and a vehicle (0.35% DMSO) and then the treated cultures were incubated for 72 h. A Matrix Wellmate dispenser (Thermo Fisher Scientific) was used to dispense CellTiter-Glo Luminescent assay reagent (10 µL per well) (Promega) into the 384-well plates. The plates were incubated for 20 min at room temperature. An Enspire microplate reader (Perkin Elmer Inc.) was used to read the luminescence. Relative cytotoxicity (%) of the compounds was determined by dividing the specific luminescence of the treated samples by the specific luminescence of the vehicle control and multiplying by 100.

### Data analysis

The concentrations of glansreginin A in different black walnut cultivars obtained from HPLC–MS/MS analysis were analyzed as a completely randomized design using PROC MIXED in SAS 9.4 (SAS Institute, Cary, NC, USA). The black walnut cultivar (treatment) was the fixed effect and replication was the random variable. Differences among the concentrations of glansreginin A in the black walnut cultivars were determined using Fisher’s LSD at *p* < 0.05.

For total antioxidant capacity, GraphPad Prism 9 (San Diego, CA, USA) was used to generate linear regression equations for each compound. Coefficients of the compound equations were compared with that of Trolox control to determine the relative total antioxidant capacity of each compound. Fold-increase over Trolox was determined by dividing the coefficient of the compound models by that of the Trolox control. For antibacterial and anticancer activities, non-linear regression analysis was performed to identify the dose–response curve for each compound using GraphPad Prism 9. Half maximal inhibitory concentrations (IC_50_ values) of each compound tested were determined from the dose–response curve for the A549 and MRC-5 cell lines.

### Ethic statement

The experimental research and collection of plant materials of this study comply with the relevant institutional, national, and international guidelines and legislation.

### Supplementary Information

Supplementary Information.

## Data Availability

All pertinent data are found in the figures and tables. Requests for data and additional information should be submitted to the corresponding author.
