# Supplementary material for: Quantification and characterization of biological activities of glansreginin A in black walnuts (Juglans nigra)
Source: Sci Rep. 2023 Nov 1;13:18860. doi: 10.1038/s41598-023-46134-8 (PMC10620390; doi:10.1038/s41598-023-46134-8)
Supplement: Supplementary file 1 — Supplementary Information. [file 41598_2023_46134_MOESM1_ESM.docx]

**Quantification and Characterization of Biological Activities of Glansreginin A in Black Walnuts (*Juglans nigra*)**

Khanh-Van Ho^1,2,3,4^, Hsien-Yeh Hsieh^1^, Anuradha Roy^5^, Sarah Foote^6^, Peter McDonald^5^, Mark V. Coggeshall^7^, Hideyuki Ito^8^, Zhentian Lei^9,10^, Lloyd W. Sumner^9,10^, George C. Stewart^11^, and Chung-Ho Lin^1*^

^1^Center for Agroforestry, School of Natural Resources, University of Missouri, Columbia, Missouri, United States

^2^Department of Chemistry, University of Missouri, Columbia, Missouri, United States

^3^Molecular Imaging and Theranostics Center, University of Missouri, Columbia, MO, United States

^4^Department of Food Technology, Can Tho University, Can Tho, Vietnam

^5^High Throughput Screening Laboratory, University of Kansas, Lawrence, Kansas, United States

^6^CEVA Biomune, Lenexa, Kansas, United States

^7^Northern Research Station, USDA Forest Service, West Lafayette, Indiana, United States

^8^Faculty of Health and Welfare Science, Department of Nutritional Science, Okayama Prefectural University, Okayama, Japan

^9^Metabolomics Center, University of Missouri, Columbia, Missouri, United States

^10^Department of Biochemistry, Bond Life Sciences Center, University of Missouri, Columbia, Missouri, United States

^11^Department of Veterinary Pathobiology, Bond Life Sciences Center, University of Missouri, Columbia, Missouri, United States

*Corresponding Author: Chung-Ho Lin, PhD

Research Professor

University of Missouri

Center for Agroforestry

203 ABNR Natural Resources Building

University of Missouri

Columbia, Missouri 65211, United States

Phone: 573-882-6283

Email: LinChu@missouri.edu

**Supplementary Information**

**Supplementary Table 1.** Antioxidant activities of glansreginin A.

| **No.** | **Compound** | **Slope (in Trolox Equivalents)** | **R square** | **Fold-increase over Trolox** | |
| --- | --- | --- | --- | --- | --- |
| 1 | Trolox | 0.00100 ± 6.5×10^-6^ | 0.999 | | 1.00 |
| 2 | Glansreginin A | 0.00012 ± 2.3×10^-6^ | 0.988 | | 0.12 |
| 3 | text - Butylhydroquinone | 0.01044 ± 5.9×10^-4^ | 0.995 | | 10.44 |
| 4 | DL - Sulforaphane | 0.00123 ± 7.0×10^-5^ | 0.999 | | 1.23 |

**Supplementary Table 2.** Half maximal inhibitory concentrations (IC_50_) of glansreginin A (µM) in A549 and MRC-5 cell lines.

| **No.** |  | **Compound** | **A549 cell line** | **MRC-5 cell line** |
| --- | --- | --- | --- | --- |
| 1 |  | Glansreginin A | >250 | >250 |
| 2 |  | Trolox | >120 | >120 |
| 3 |  | text - Butylhydroquinone | 139.20 | 108.50 |
| 4 |  | DL-Sulforaphane | 16.87 | 9.27 |
